# The *Plasmodium falciparum* Erythrocyte Invasion Ligand Pfrh4 as a Target of Functional and Protective Human Antibodies against Malaria

**DOI:** 10.1371/journal.pone.0045253

**Published:** 2012-09-20

**Authors:** Linda Reiling, Jack S. Richards, Freya J. I. Fowkes, Danny W. Wilson, Watcharee Chokejindachai, Alyssa E. Barry, Wai-Hong Tham, Janine Stubbs, Christine Langer, John Donelson, Pascal Michon, Livingstone Tavul, Brendan S. Crabb, Peter M. Siba, Alan F. Cowman, Ivo Mueller, James G. Beeson

**Affiliations:** 1 Burnet Institute of Medical Research and Public Health, Victoria, Australia; 2 Walter and Eliza Hall Institute of Medical Research, Victoria, Australia; 3 Faculty of Tropical Medicine, Mahidol University, Bangkok, Thailand; 4 Department of Medical Biology, University of Melbourne, Victoria, Australia; 5 Department of Biochemistry, University of Iowa, Iowa City, Iowa, United States of America; 6 Papua New Guinea Institute of Medical Research, Madang, Papua New Guinea; 7 Barcelona Center for International Health Research, Barcelona, Spain; 8 Melbourne School of Population Health, University of Melbourne, Victoria, Australia; 9 Center for Emerging and Neglected Infectious Diseases, Mahidol University, Nakorn Pathom, Thailand; London School of Hygiene and Tropical Medicine, United Kingdom

## Abstract

**Background:**

Acquired antibodies are important in human immunity to malaria, but key targets remain largely unknown. *Plasmodium falciparum* reticulocyte-binding-homologue-4 (PfRh4) is important for invasion of human erythrocytes and may therefore be a target of protective immunity.

**Methods:**

IgG and IgG subclass-specific responses against different regions of PfRh4 were determined in a longitudinal cohort of 206 children in Papua New Guinea (PNG). Human PfRh4 antibodies were tested for functional invasion-inhibitory activity, and expression of PfRh4 by *P. falciparum* isolates and sequence polymorphisms were determined.

**Results:**

Antibodies to PfRh4 were acquired by children exposed to P. falciparum malaria, were predominantly comprised of IgG1 and IgG3 subclasses, and were associated with increasing age and active parasitemia. High levels of antibodies, particularly IgG3, were strongly predictive of protection against clinical malaria and high-density parasitemia. Human affinity-purified antibodies to the binding region of PfRh4 effectively inhibited erythrocyte invasion by *P. falciparum* merozoites and antibody levels in protected children were at functionally-active concentrations. Although expression of PfRh4 can vary, PfRh4 protein was expressed by most isolates derived from the cohort and showed limited sequence polymorphism.

**Conclusions:**

Evidence suggests that PfRh4 is a target of antibodies that contribute to protective immunity to malaria by inhibiting erythrocyte invasion and preventing high density parasitemia. These findings advance our understanding of the targets and mechanisms of human immunity and evaluating the potential of PfRh4 as a component of candidate malaria vaccines.

## Introduction

Malaria due to *Plasmodium falciparum* remains a major global health burden and a leading cause of death worldwide among children under five [1], [2]. Increasing drug resistance, including emerging resistance to the artemisinin drugs, and the declining efficacy of vector control interventions in some populations make the development of effective malaria vaccines an urgent priority. During blood-stage infection, *P. falciparum* merozoites invade erythrocytes, mediated by the release of invasion ligands from apical organelles that interact with receptors on the erythrocyte surface [3], [4]. The repertoire of invasion ligands includes two major families, the *P. falciparum* reticulocyte-binding homologues (PfRh), and erythrocyte binding antigens (EBAs) [3], [4]. The ability of *P. falciparum* to vary the expression and/or use of EBA and PfRh proteins enables the use of alternate invasion pathways [5], [6], facilitating immune evasion that enables *P. falciparum* to cause repeated and chronic infections [7]. Invasion pathways can be broadly classified into two main pathways, sialic acid (SA)-dependent invasion and SA-independent invasion.

The PfRh ligands are located in the rhoptries of merozoites and include PfRh1, PfRh2a, PfRh2b, PfRh4 and PfRh5 [3], [6], [8], [9], [10]. PfRh4 binds to complement receptor 1 and is essential for SA-independent invasion [6], [11], [12], [13], whereas the EBAs and PfRh1 are important for SA-dependent invasion [8], [14], [15], [16], [17], [18]. Expression of PfRh4 varies among isolates, but knowledge on the extent of variation and the frequency of expression of PfRh4 by isolates is limited. There are data on expression of the *Pfrh4* gene by isolates from infected individuals in Africa [19], [20], and data on PfRh4 expression by a small number of laboratory-adapted isolates [6], [11], [21]; however, there are presently no data on expression of PfRh4 protein by clinical isolates, or data from populations outside Africa.

Protective immunity to malaria eventually develops after repeated exposure, and is thought to prevent disease by controlling blood-stage parasitemia [22], [23], [24], [25]. Despite an expanding knowledge of the genomics and proteomics of *P. falciparum*, few antigens have been studied as immune targets in humans [22], [23] and there is a paucity of data on functional immune responses to specific antigens. These gaps have restricted our knowledge of immunity and impeded progress towards developing effective vaccines. Antibodies to erythrocyte invasion ligands may act by directly inhibiting parasite replication, possibly also through antibody dependent cellular inhibition, and opsonization of merozoites for phagocytosis [7], [22], [26], [27], [28], [29], [30], [31]. Very little is known about immune responses to PfRh proteins. An initial study in Kenya reported that antibodies to PfRh2 and PfRh4 were acquired in an age-dependent manner, reflecting the acquisition of immunity in the population, and antibodies to PfRh2 were associated with protective immunity in a prospective study of children [7], [30]. In light of the important role of PfRh4 in invasion, we have evaluated PfRh4 as a target of human immunity.

## Materials and Methods

### Protein Expression

**Figure 1:**
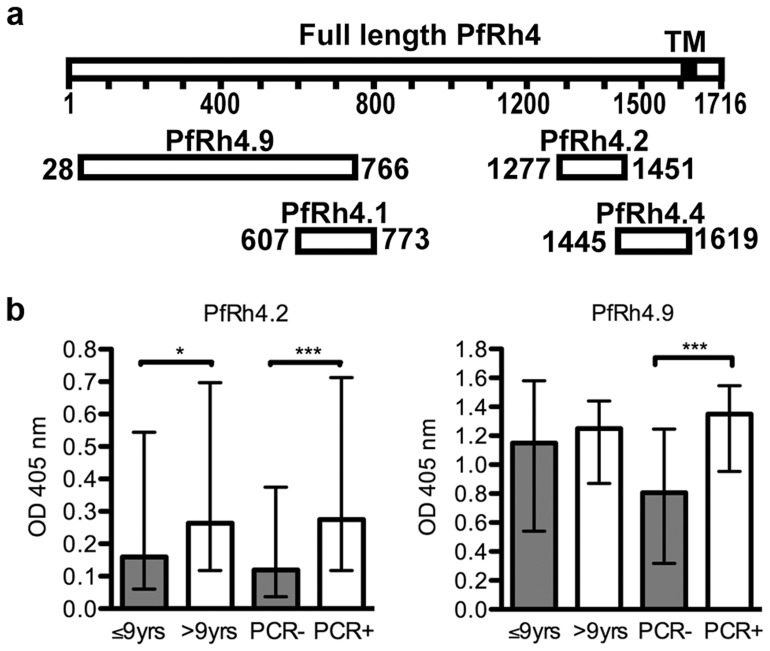
PfRh4 is a target of human antibodies. (a) Schematic representation of the PfRh4 recombinant proteins used in this study, relative to full length PfRh4. TM: transmembrane domain. Numbers represent amino acids. (b) IgG reactivity to PfRh4 recombinant proteins measured by ELISA. Results are shown as median OD and interquartile ranges by age group (≤ or >9 years of age) or parasitemic status (PCR- or PCR+) for PfRh4.2 (left panel) and PfRh4.9 (right panel).

Recombinant protein PfRh4.9 (amino acids [aa] 28–766; Figure 1) contains the erythrocyte binding region of PfRh4, and the expression, purification, and functional binding activity of the protein has been previously reported [32]. In brief, it was expressed as a (His)_6_ fusion protein, and subsequently purified on a Ni-nitrilotriacetic acid column followed by a Superdex gel filtration column. Recombinant proteins PfRh4.2 (amino acids aa 1277–1451), PfRh4.1 (aa 607–773) and PfRh4.4 (aa1445–1619) were expressed as GST fusion proteins and purified on a glutathione agarose column (Sigma-Aldrich, Sydney, Australia), and then dialysed against PBS, as previously described [6]. The PfRh4 sequence used was from the reference isolate 3D7.

### ELISA

IgG and IgG subclass reactivity to recombinant proteins were measured by ELISA using established methods [25], [30], [31]. Sera were tested at 1/500. Samples were classified as antibody positive if the OD>mean+3SD of reactivity observed with non-exposed sera. Reactivity of antibodies among samples to GST was negligible.

### Study Population and Ethics Approval

Plasma samples were obtained from a prospective treatment re-infection study of 206 children from Mugil, Madang Province, Papua New Guinea (PNG) described in detail elsewhere [33]; median age was 9.3 years (range: 5–14). At enrolment the prevalence of *P. falciparum* was 67.5% (n = 139) by PCR and 40.3% (n = 83) by light microscopy (the geometric mean parasite density was 361 parasites/µl (95% CI, 240–544). After enrolment, all children received 7 days of artesunate orally. Children were reviewed 2-weekly for 6 months for symptomatic illness and parasitemia by PCR and microscopy, and by passive case detection. A clinical episode of *P.falciparum* malaria was defined as fever and *P.falciparum* parasitemia >5000/µl. During the follow-up period, 80 children experienced clinical malaria, 196 had re-infection by PCR, and 180 had re-infection detected by light microscopy. Samples used were those taken at enrolment, prior to artesunate treatment. Sera were also obtained from anonymous Australian residents as controls. Ethical approval for this study was obtained from the Medical Research Advisory Committee, PNG and the Human Research Ethics Committees of the Walter and Eliza Hall Institute and Alfred Hospital, Australia. Written informed consent was obtained from subjects and their guardians.

### Statistical Analysis

Statistical analysis was performed using STATA 9.2 (STATACorp, College Station, Texas, USA). Differences in seroprevalence and IgG levels between categorical variables were assessed using chi-square tests or Kruskal Wallis tests, respectively. To determine the association between antibody levels and subsequent episodes of *P. falciparum* infection and symptomatic malaria, children were stratified into three equal groups (tertiles) reflecting low (including those who were classified as sero-negative), medium and high responders according to OD values for each antigen; the risk of malaria and parasitemia was compared between responder groups, as used previously [30], [31]. Although some children had multiple episodes of parasitemia or malaria, analysis included time to first re-infection or first symptomatic episode only. All children were treated with 7 days artesunate, orally, at enrolment to clear existing parasitemia, and treatment efficacy was 91.4% (as determined by PCR) [33]. Treatment failures (n = 12) were differentiated from re-infection by genotyping of *msp2*, and were excluded from the analysis. The Cox proportional hazards model was used to calculate hazard ratios for risk of malaria between antibody responder groups. For antibody variables that showed non-proportional hazards, an interaction term between the antibody response and time (3 categories: t = 0–100, t = 100–150 and t>150 days) was included in the analysis. A range of demographic, clinical and biological variables were assessed as potential confounders of associations between antibodies and malaria outcomes. Only host age and location of residence were identified as being significantly associated with antibodies and malaria outcomes [25], [33]. Of note, parasitemia status at enrolment and red blood cell polymorphisms were not associated with malaria outcomes [33], [34]. Therefore, multivariate analysis was used to calculate adjusted hazard ratios using covariates of age and location. Age was used as binary variable (<9 years, ≥9 years), as previous studies indicated that this stratification was the most informative approach for assessing the effect of age [33]. Additional age groupings with narrower age ranges were explored in the multivariate analysis; however, results using these groupings in the analysis were not different to using age as a dichotomous variable. Adjusting for host gender did not influence protective associations. Some previous studies have stratified analyses based on the presence or absence of parasitemia at baseline [35] because it was found to influence antibody associations. However, in our study, parasitemia at enrolment was not significantly associated with malaria outcomes and including parasitemia status in the analysis did not affect the multivariate regression models. There was no significant difference in PfRh4 antibodies according to gender. Furthermore, adjustments for gender in multivariate models did not significantly alter model outputs; therefore, gender was not included in the final model. Recombinant protein constructs PfRh4.1, PfRh4.2, and PfRh4.4 were GST-fusion proteins; reactivity to GST was very low, and was not associated with risk of malaria, and deducting GST reactivity from the reactivity to Rh4-GST fusion proteins did not affect analysis outcomes. Reporting of study outcomes followed the MIOS guidelines for malaria immuno-epidemiology observational studies [22].

### Immunofluorescence Microscopy (IFAs) and Western Blots for PfRh4 Expression

Using blood samples collected at enrolment, erythrocytes were separated from PBMCs by density centrifugation over a Ficoll-Paque gradient (Ficoll-Paque Plus, Amersham, [36]. Parasites were cultured for 36 hours until mainly segmented schizonts were prevalent, washed twice in PBS, thin smears made, air dried then fixed in methanol (−20°C) for 2 minutes. Slides were treated for 10 minutes with 0.1% Triton X100 prior to blocking (30 minutes, 3% BSA), and then sequentially incubated with monoclonal anti-PfRh4 (1∶50) and polyclonal rabbit antibodies to *P. falciparum* rhoptry neck protein PfRON4 (1∶1000), anti-mouse-Alexa 594 (1∶500) and anti-rabbit-Alexa 488 (1∶750). A DAPI containing mounting medium was added (VectaShield, Vector Labs). An isolate was considered positive for PfRh4 expression only if at least 3 DAPI-positive schizonts stained positive with anti-PfRh4. For western blots, protein extracts of *P. falciparum* schizonts were obtained by Saponin lysis of a synchronized parasite culture [7]. Schizont pellets were boiled in reducing sample buffer. SDS PAGE and Western Blot were performed using established procedures. Monoclonal anti-PfRh4 antibody was used 1/500, rabbit anti-Hsp70 1/500, anti-mouse-HRP conjugate 1/1000 and anti-rabbit-HRP conjugate 1/25000, all in 1% casein. The specificity of the PfRh4 MAb has been previously established by demonstrating that it labels a protein of expected size in parasites that are known to express *pfrh*4 gene, and does not label a similar protein in parasites in which the *pfrh4* gene has been disrupted [32]. Isolates used included 3D7, HB3, W2mefΔPfRh4 [6], HCS3 [37], XIE, Pf2004, Pf2006 [38].

### Affinity-purification of Human Antibodies

Serum samples from adults and children in the study population were screened for antibodies to PfRh4.2 and PfRh4.9 by ELISA and a pool was made of ELISA-positive samples. Recombinant antigen (PfRh4.2 or PfRh4.9) was coupled to CNBR activated Sepharose (GE Healthcare). Pooled serum was filtered to remove lipids/debris before incubating with the antigen-coupled resin overnight (4°C). Bound antibodies were eluted in 10× 1 ml steps using 0.1 M Glycine pH 2.4, and pH adjusted with 30 µl Tris pH 8.8 (3 M) and 20 µl NaCl (5 M). Eluates were pooled, dialysed against PBS, filter-sterilized, and concentrated using Ultracel columns (Millipore, MWCO 50 kDa). Concentration was determined by densitometry using known concentrations of human IgG. After affinity purification, samples were tested for IgG reactivity to a panel of recombinant merozoite proteins to validate the procedure. This demonstrated that the PfRh4-affinity-purified antibody fraction was enriched for PfRh4.9 reactivity, or PfRh4.2 reactivity, and the eluates from the column showed a marked depletion of reactivity (data not shown). There was no depletion of antibodies to other merozoite proteins.

### Growth Inhibition Assays

Antibodies were tested for *in vitro* inhibition of growth over two cycles of replication, as described previously [39], [40], using the 3D7 parasite isolate. Neuraminidase-treated erythrocytes were used in some assays [7]. All samples were tested in duplicate and results were expressed as a percent growth in a PBS control.

### Sequence Analysis of PfRh4

Regions encoding PfRh4.2 and PfRh4.9 were amplified and sequenced (accession numbers HE610476–610500) using *P. falciparum* genomic DNA extracted from infected blood samples from children in the cohort or samples from other children and adults in the same township as described [30], [41], [42]. To sequence PfRh4.2, oligonucleotide primers PfRh4-A3-fwd 5′ caaataaaaaatgtcagtgatgtattcacac 3′ and PfRh4-A3-rev 5′ gacatttgtcttgtctcctatggtg 3′ were used to amplify a 525 bp fragment coding for PfRh4.2, as described in [30]. PfRh4.9 was amplified using a hemi-nested PCR strategy [42]. In the primary PCR reaction, two overlapping fragments were amplified (nucleotides 150–1423 and 730–2010 relative to the 3D7 reference sequence, AF432854) using primers: Rh4-F1 (5′aaaacacaccatacgaacaa 3′)/Rh4-R2 (5′ tggataaacatatatcatcggta 3′) and Rh4-F2 (5′ acacaaacaagtttgaacataa 3′)/Rh4_R3 (5′ ttgttgaatagttttgtgtgtaaa 3′). In the secondary PCR reaction, three fragments were amplified (nucleotides 150–826, 730–1423 and 1340–2010) from the corresponding primary PCR product using primers Rh4_F1/Rh4_R1 (5′ tgttaaatgacaatcggaat 3′), Rh4-F2/Rh4-R2 and Rh4F3 (5′ tctcattcgataaaaataaatca 3′). High quality PCR products were purified and sequenced directly. Only polymorphisms found in two independent PCR products were scored. If there was a discrepancy between the two PCR products, a third PCR product was sequenced to confirm the correct sequence. Using BLAST, PhRh4.2 and PfRh4.9 sequences from further isolates were identified in the Broad and Sanger Institutes’ database including Pf_Senegal (Senegal), RO33 and Ghana (Ghana), RAJ116 and ICH-CR14 (India), HB3 (Honduras), IT and 7G8 (Brazil), D10 (PNG), 3D7 (unknown origin) and Dd2 (SE Asia).

## Results

### Antibodies to PfRh4.2 and PfRh4.9 are Associated with Age and Concurrent Parasitemia

We measured antibodies in all cohort samples to various regions of PfRh4 expressed as recombinant proteins (Figure 1a). PfRh4.9, expressed and purified as a his-tagged protein, was selected because it contains the erythrocyte-binding region [32]. PfRh4.2 (GST-tagged) is located within the C-terminal region [6], and is known to be recognised by human antibodies [7]. Two additional proteins were expressed as GST-fusion proteins and evaluated; these were PfRh4.4 and PfRh4.1. The prevalence of IgG against PfRh4.2 and PfRh4.9 was high (94–96%) in the cohort (Table 1), whereas there was no reactivity among samples from unexposed Australian residents. The prevalence antibodies to PfRh4.2 and PfRh4.9 was significantly higher in older children (p<0.01 and p<0.001, respectively), and among children who were parasitemic at time of sample collection (p = 0.042 and p<0.001 for PfRh4.2 and PfRh4.9, respectively), consistent with the specific acquisition of antibodies from *P. falciparum* exposure. Antibodies levels also tended to be higher among older children and those who had active parasitemia (Figure 1b). There was a significant correlation between antibodies against PfRh4.2 and PfRh4.9 (p<0.001; Table 2), and antibodies to PfRh4.2 and PfRh4.9 were significantly correlated with antibodies to other merozoite antigens, such as EBA175 (p<0.001), suggesting that antibodies to PfRh4 are co-acquired with other responses to merozoite antigens. Prior studies have shown that antibodies generated in rabbits against recombinant PfRh4.2 and PfRh4.9 reacted specifically with native PfRh4 [6], [32], indicating that epitopes expressed by native proteins are represented on the recombinant proteins we used in ELISAs. In contrast, antibodies to the two other constructs, PfRh4.1 and PfRh4.4, were generally low and antibodies were not significantly associated with age or parasitemia status. This suggests that these regions may not be naturally immunogenic, or contain no or few antibody epitopes. Alternatively, the recombinant proteins may not be appropriately folded to present epitopes; however, in the absence of knowledge of the structure of PfRh4 this is difficult to assess.

**Table 1:** Prevalence and levels of antibodies to PfRh4 antigens.

	All	Age^2^			Enrolment *P. falciparum* parasitemic status^3^		
		≤9.0 yrs	>9.0 yrs	P^4^	PCR−	PCR+	P^4^
	n = 206	n = 91	n = 115		n = 67	n = 139	
**PfRh4.2**							
**IgG**							
Seropositive^1^	195	82	113	0.01	60	135	0.042
%	94.7	90.1	98.2		90	97	
Median OD	0.24	0.16	0.26	0.01	0.12	0.28	0.0001
[IQR]	[0.08–0.64]	[0.1–0.54]	[0.12–0.7]		[0.04–0.38]	[0.12–0.71]	
**PfRh4.9**	**n = 203**						
**IgG**							
Seropositive^1^	195	82	113	0.001	58	137	<0.0001
%	96.1	91.1	100		87.7	100	
Median OD	1.23	1.15	1.25	0.6	0.81	1.35	<0.0001
[IQR]	[0.74–1.51]	[0.54–1.58]	[0.87–1.44]		[0.32–1.25]	[0.95–1.55]	
**PfRh4.4**							
**IgG**							
Seropositive^1^	31	13	18	0.785	8	23	0.386
%	15.1	14.3	15.7		12.0	16.6	0.386
Median OD	0.12	0.12	0.12	0.571	0.12	0.12	0.995
[IQR]	[0.08–0.19]	[0.08–0.19]	[0.08–0.2]		[0.07–0.2]	[0.08–0.19]	0.995
**PfRh4.1**							
**IgG**							
Seropositive^1^	78	34	44	0.895	24	54	0.675
%	37.9	37.4	38.3		35.8	38.9	
Median OD	0.15	0.15	0.15	0.544	0.15	0.15	0.732
[IQR]	[0.1–0.22]	[0.08–0.21]	[0.1–0.22]		[0.09–0.23]	[0.1–0.22]	
**PfRh4.2**							
**subclasses**							
**IgG1**							
Seropositive^1^	30	10	20	0.2	5	25	0.045
%	14.6%	11.0%	17.4%		7.5%	18.0%	
Median OD	0.06	0.04	0.06	0.36	0.04	0.06	0.056
[IQR]	[0.01–0.12]	[0.01–0.11]	[0.01–0.13]		[0–0.09]	[0.02–0.13]	
**IgG2**							
Seropositive^1^	17	10	7	0.2	3	14	0.172
%	8.3%	11%	6.1%		4.5%	10.1%	
Median OD	0.01	0	0.01	0.32	0	0	0.169
[IQR]	[0–0.01]	[0–0.01]	[0–0.02]		[0–0.11]	[0–0.02]	
**IgG3**							
Seropositive^1^	182	77	105	0.06	50	132	0.003
%	93.3%	89.5%	96.3%		84.8%	97.1%	
Median OD	0.19	0.13	0.25	0.01	0.1	0.25	<0.0001
[IQR]	[0.06–0.63]	[0.04–0.44]	[0.07–0.74]		[0.02–0.25]	[0.07–0.84]	<0.0001
**IgG4**							
Seropositive^1^	5	2	3	1.0	2	3	0.661
%	2.4%	2.2%	2.6%		3.0%	2.16%	
Median OD	0	0	0	0.15	0	0	0.138
[IQR]	[0–0.01]	[0–0.02]	[0–0.01]		[0–0.01]	[0–0.02]	
**PfRh4.9** **subclasses**							
**IgG1**							
Seropositive^1^	153	63	90	0.14	36	117	<0.0001
%	74.3%	69.2%	78.3%		53.7%	84.2%	
Median OD	0.41	0.39	0.44	0.25	0.21	0.54	<0.0001
[IQR]	[0.14–0.78]	[0.10–0.86]	[0.20–0.73]		[0.04–0.48]	[0.23–0.84]	
**IgG2**							
Seropositive^1^	0	0	0	–	0	0	–
%	0%	0%	0%		0%	0%	
Median OD	0	0	0	0.22	0	0	0.111
[IQR]	[0–0.02]	[0–0.02]	[0–0.02]		[0–0.01]	[0–0.02]	
**IgG3**							
Seropositive^1^	75	17	58	<0.0001	12	63	<0.0001
%	36.4%	18.7%	50.4%		17.9%	45.3%	
Median OD	0.07	0.03	0.13	<0.0001	0.03	0.11	<0.0001
[IQR]	[0.02–0.2]	[0.01–0.86]	[0.05–0.27]		[0.01–0.07]	[0.03–0.24]	
**IgG4**							
Seropositive^1^	15	5	10	0.38	4	11	0.778
%	7.3%	5.5%	8.7%		6.0%	7.9%	
Median OD	0	0	0	0.3	0	0	0.130
[IQR]	[0–0.02]	[0–0.02]	[0–0.03]		[0–0.01]	[0–0.03]	

**Notes:**

1 Seropositive (number of seropositive individuals) and percentage (%) of seropositive individuals were defined by IgG reactivity that was higher than the mean plus three standard deviations of control sera (unexposed donors) measured by ELISA. Median optical densities (OD) are displayed; [IQR] - interquartile range.

2 Age: ≤9.0 yrs indicates individuals younger than 9 years of age; >9.0 yrs indicates individuals older than 9 years of age.

3 Parasitemic status: PCR-, indicates *P. falciparum* was not detected by PCR; PCR+, indicates *P. falciparum* was detected by PCR.

4 P values were calculated using the chi-squared test or Fisher’s exact test for comparisons of seroprevalence, or using the Kruskal-Wallis test for comparisons of antibody level.

**Table 2:** Correlation between IgG and IgG subclass responses to PfRh4 proteins.

	PfRh4.2						PfRh4.9					PfRh4.4
		IgG	IgG1	IgG2	IgG3	IgG4	IgG	IgG1	IgG2	IgG3	IgG4	IgG
PfRh4.2	IgG											
	IgG1	0.64*										
	IgG2	0.11	**0.2**									
	IgG3	0.81*	0.42*	−0.01								
	IgG4	**0.17**	**0.15**	−0.13	**0.2**							
PfRh4.9	IgG	0.44*	**0.24**	−0.002	0.42*	0.12						
	IgG1	0.43*	**0.22**	0.012	0.4*	0.09	0.94*					
	IgG2	**0.16**	0.09	0.33*	0.07	−0.09	0.28*	**0.24**				
	IgG3	0.54*	0.33*	0.08	0.52*	0.08	0.36*	0.36*	**0.2**			
	IgG4	0.11	0.09	0.1	0.02	0.12	**0.21**	**0.2**	0.32*	**0.19**		
PfRh4.4	IgG	0.34*	**0.24**	0.1	**0.27**	0.13	0.07	0.08	−0.02	**0.16**	0.02	
PfRh4.1	IgG	0.45*	0.32*	0.03	0.35*	0.05	0.34*	0.36*	−0.02	**0.2**	−0.03	0.53*

Notes: Correlation coefficients are Spearman’s rho.*: p<0.0001. Bold: p<0.05. Others: not significant. IgG: total IgG. Antibodies measured by ELISA.

### IgG Subclass Responses are Predominantly IgG1 and IgG3

The nature of IgG subclasses can influence function [43], and we found that IgG1 and IgG3, known as cytophilic subclasses, formed the predominant responses (Table 1). Most children were seropositive for PfRh4.2 specific IgG3 (93.3%), whereas seropositivity for PfRh4.2 specific IgG1, IgG2 and IgG4 was much lower (14.6%, 8.3%, and 2.4%, respectively). IgG3 to PfRh4.2 was significantly higher among older children (p = 0.01–0.06) and children with active parasitemia (p<0.01). IgG1 was the most prevalent response against PfRh4.9 (74.3%) and was significantly higher in parasitemic children (p<0.001), but was not associated with age. The prevalence of PfRh4.9 specific IgG3 was 36.4%, and was strongly associated with age (p<0.0001) and parasitemic status (p<0.0001). There was no PfRh4.9 specific IgG2 and very little IgG4 detected.

### Antibodies to PfRh4 are Associated with a Reduced Risk of Clinical Malaria and High-density Parasitemia

The design and longitudinal nature of the cohort study, incorporating anti-malarial treatment at enrolment and active follow-up with the application of sensitive molecular methods to detect *P. falciparum* infections, enabled us to investigate relationships between PfRh4 specific antibodies and re-infection, parasitemia of different densities and symptomatic malaria. A range of factors were assessed for potential confounding of antibody associations with malaria outcomes; of these, only host age and residential location were identified as possible confounders [33], [34]. Total IgG, IgG1 and IgG3 against PfRh4.2 were all significantly associated with protection from clinical malaria (defined as parasitemia >5000 parasites/µl plus fever) when comparing high and low antibody responders (HR 0.23, p<0.0001; 0.36, p = 0.001 and 0.4, p = 0.003, respectively, Table 3). Importantly, these associations remained significant after adjustment for age and location of residence (adjusted HR [aHR] 0.29, p = 0.001; 0.39, p = 0.004 and 0.51, p = 0.04, respectively). The association between antibodies to PfRh4 and age was modest, and adjusting for age, on its own, in survival analyses did not significantly alter the hazard ratios (using age as a dichotomous variable of <9 and >9 years, or using multiple age categories). Some previous studies have reported hazard ratios stratified for parasitemia status at baseline because of an interaction between antibodies, parasitemia and malaria risk. Although antibodies were higher among those with current parasitemia in this study, parasitemia at baseline was not significantly associated with risk of malaria. Therefore, parasitemia was not included as a confounding variable in our final analyses, and survival analyses were not stratified by baseline parasitemia status; adjusting for parasitemia at enrolment did not affect hazard ratios. High levels of IgG and IgG3 against PfRh4.2 were also significantly associated with a reduced risk of high-density parasitemia (defined as >5000 parasites/ul; aHR: 0.51, p = 0.028, and aHR: 0.52, p = 0.027, respectively; Supporting data, Table S1).

**Table 3:** Associations between antibodies and risk of clinical malaria.

Antigen		uHR MvL [95% CI]	p	uHR HvL [95% CI]	p	aHR MvL [95% CI]	p	aHR HvL [95% CI]	p
PfRh4.2	IgG	0.56[0.33–0.95]	0.03	0.23[0.12–0.47]	<0.0001	0.66[0.38–1.14]	0.138	0.29[0.14–0.58]	0.001
	IgG1	0.58[0.33–1.0]	0.048	0.36[0.2–0.67]	0.001	0.64[0.36–1.12]	0.118	0.39[0.21–0.74]	0.004
	IgG3	0.53[0.3–0.92]	0.023	0.4[0.22–0.73]	0.003	0.64[0.36–1.13]	0.122	0.52[0.28–0.98]	0.043
PfRh4.9	IgG	0.62[0.26–1.5]	0.293	0.68[0.29–1.58]	0.368	0.71[0.3–1.73]	0.454	0.73[0.31–1.71]	0.462
	IgG1	0.41[0.16–1.06]	0.065	0.73[0.33–1.65]	0.451m	0.49[1.88–1.29]	0.148	0.83[0.37–1.88]	0.662
	IgG3	0.11[0.03–0.36]	<0.0001	0.14[0.05–0.41]	<0.0001	0.12[0.04–0.4]	0.001	0.18[0.06–0.54]	0.002

Notes: Study participants (n = 206) were stratified into 3 equal groups according to low, medium or high levels of antigen-specific antibodies. Hazard ratios were calculated comparing high versus low levels of antibodies (HvL) and medium versus low levels (MvL) of antibodies for the risk of symptomatic malaria over 6 months of follow-up; analysis was based on first episode only. Unadjusted hazard ratios (uHR), adjusted (age-adjusted and location-adjusted) hazard ratios hazard ratios (aHR) and 95% confidence intervals [95% CI] were calculated. During the follow-up period, 80 children experienced at least one episode of clinical malaria, and 196 were re-infected (as detected by PCR).

High PfRh4.9-specific IgG3 was strongly associated with a reduced risk of clinical malaria (HR: 0.14, p<0.0001; aHR: 0.18, p = 0.002); this was the strongest association with protection seen in this study. PfRh4.9 specific IgG3 was also predictive of protection against high-density parasitemia (Table S1). Importantly, the strength and significance of the association remained high after adjusting for age and residential location, and were not affected by adjusting for parasitemia at enrolment. In contrast, IgG1 was not significantly associated with protection. Antibodies to PfRh4.1 and PfRh4.4 were low and not associated with protection (data not shown). Due to the low prevalence of IgG2 and IgG4 to PfRh4, associations with protection were not examined. None of the examined antibody levels was associated with protection from re-infection *per se*.

The protective association for IgG3 to the PfRh4.9 recombinant protein (which contains the erythrocyte-binding region) was stronger than responses or the PfRh4.2 recombinant protein. To explore this further, we included IgG3 responses to PfRh4.9 and PfRh4.2 in multivariate analysis using the Cox proportional hazards model. The association between IgG3 to PfRh4.9 and reduced risk of malaria was unchanged and strongly associated with protection, whereas the association between IgG3 to PfRh4.2 and protection was weaker and non-significant. This may suggests that IgG3 to PfRh4.9 is the more important response in contributing to protection from malaria in this cohort. Because of a significant correlation between responses, interaction terms could not be explored to investigate any potential synergistic or additive effects of different antibody responses.

### Human Antibodies Against PfRh4 Inhibit Invasion of Erythrocytes

**Figure 2:**
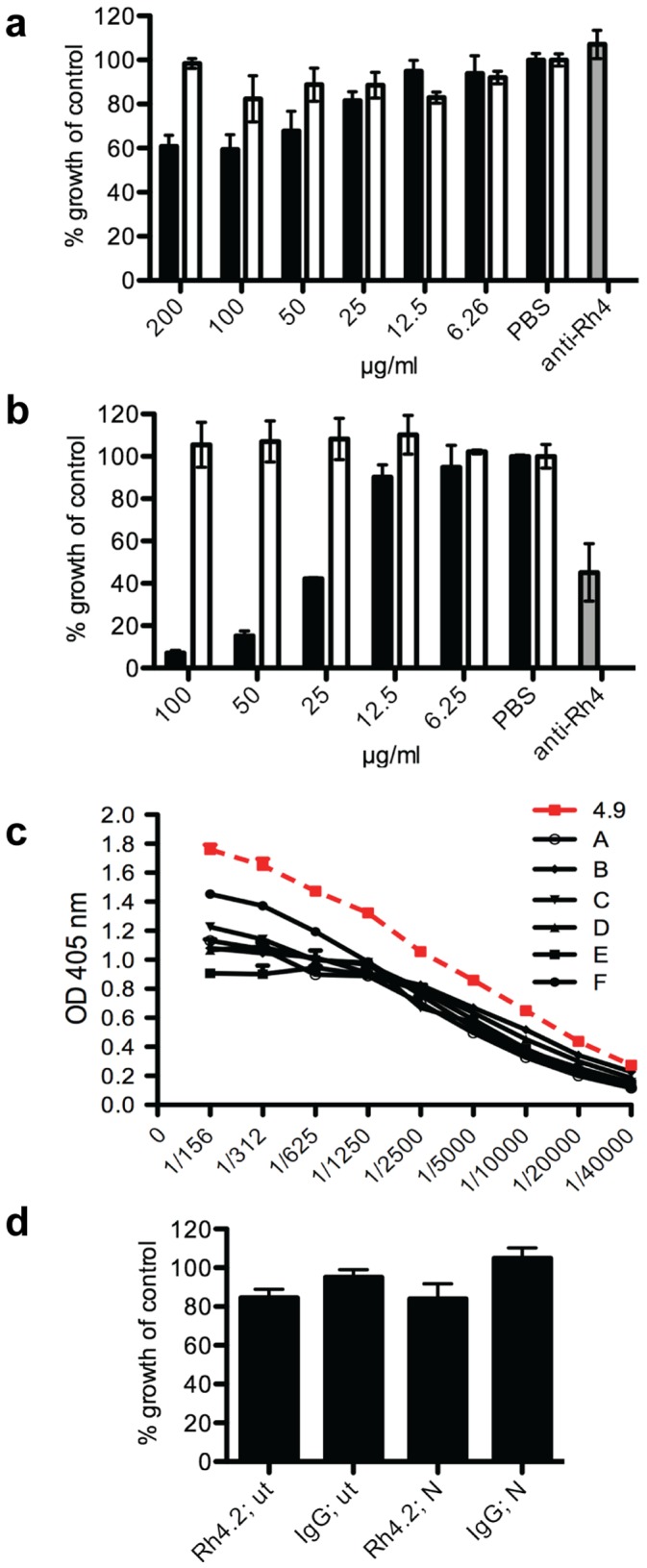
Inhibition of erythrocyte invasion by affinity-purified human PfRh4 antibodies. (a) Concentrationdependent inhibition of invasion by affinity-purified human antibodies to recombinant PfRh4.9. Increasing concentration of PfRh4.9 antibody (black bars) resulted in increased invasion inhibition into untreated erythrocytes, whereas varying concentrations of human non-immune IgG had no effect (white bars). (b) Concentration-dependent invasion inhibition by PfRh4.9 antibodies into neuraminidase treated erythrocytes. Results for (a) and (b) show means from 2 independent experiments, each run in duplicate, and are expressed as % growth compared to PBS control. Error bars represent the SEM between all 4 values. Anti-Rh4.9 rabbit antibodies, a known inhibitor of invasion, was used as a control (grey bars). (c) ELISA results plotted as OD at 405 nm (y-axis) to compare concentrations of affinity purified PfRh4.9 antibodies and high responders (samples A–F) from the Mugil cohort. Different dilutions as indicated on the x-axis have been tested on recombinant PfRh4.9. (d) Invasion inhibition mediated by anti-PfRh4.2 antibodies (at 300 µg/ml) in relation to invasion inhibition mediated by non-specific human IgG (250 µg/ml). Results represent the mean from two independent experiments, each sample tested in duplicate. Error bars represent the SEM; ut: untreated erythrocytes, N: Neuraminidase treated erythrocytes.

To assess the functional activity of PfRh4 antibodies, we affinity purified human anti-PfRh4.9 and PfRh4.2 antibodies and tested them for *P. falciparum* growth-inhibitory activity *in vitro*. Neuraminidase treatment of erythrocytes was used to inhibit the use of alternate invasion pathways and force merozoites to use PfRh4 during invasion [6], [12]. Purified PfRh4.9 antibodies inhibited invasion into untreated (Figure 2a) and neuraminidase-treated erythrocytes (Figure 2b) in a concentration-dependent manner. Inhibition of invasion was much stronger in neuraminidase treated erythrocytes, with an IC50 of 23 µg/ml (invasion into untreated cells was inhibited by 40% at 100 µg/ml). The greater inhibition of invasion into neuraminidase-treated cells reflects the reliance on PfRh4 by merozoites during invasion, and points to the specificity of the affinity-purified PfRh4 antibodies. To determine whether PfRh4.9 antibodies were present in protected children at sufficiently high levels to inhibit invasion, we directly compared the reactivity of antibodies among high responders (defined as the upper tertile) from the cohort with the reactivity of the affinity-purified invasion-inhibitory antibodies using ELISA (Figure 2c); results suggested that children did have sufficiently high enough concentrations for functional activity. IgG reactivity for the affinity-purified PfRh4.9 was 2–4 times higher than that seen for high-responder children; since the purified IgG inhibited growth by 50% by a 1/54 dilution (23.3 µg/ml), and by 75% at a 1/42 dilution (30.1 µg/ml), the children’s samples would be expected to inhibit by similar amounts at a 1/10–1/27 dilution. In contrast, human antibodies against PfRh4.2 (tested at a maximum concentration of 300 µg/ml) did not substantially inhibit invasion into either untreated or neuraminidase treated erythrocytes (Figure 2d).

### PfRh4 Expression in Isolates from Infected Children

**Figure 3:**
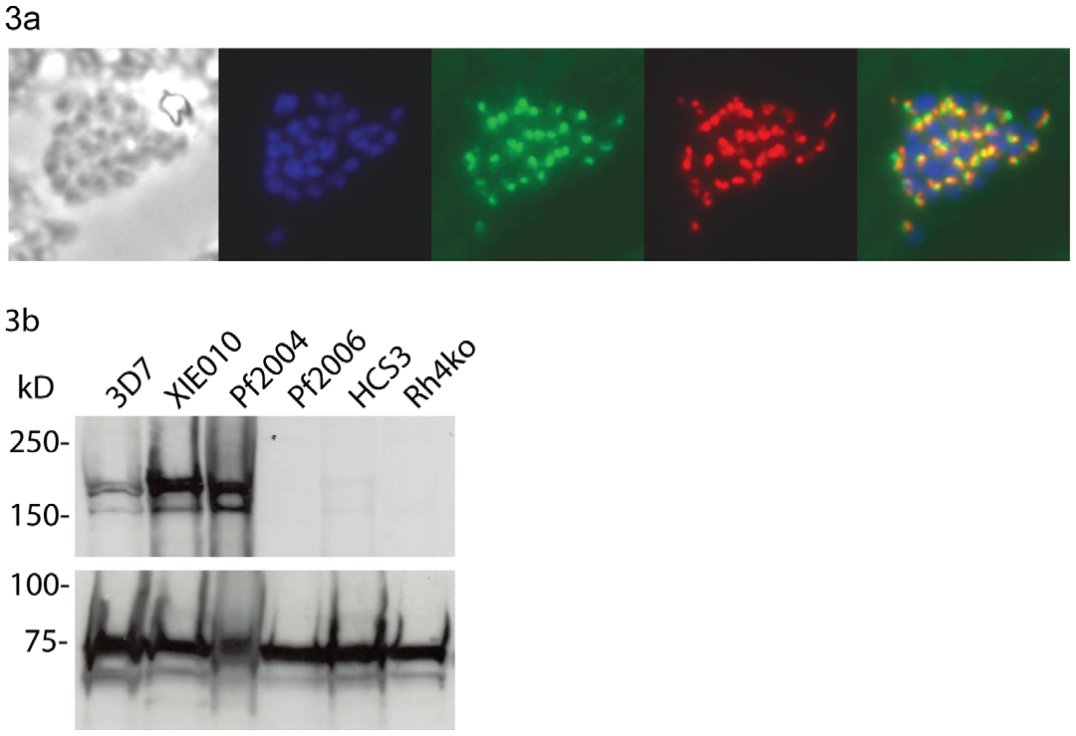
Expression of PfRh4 in clinical isolates and culture-adapted strains. (a) A representative example of a schizont from a clinical *P. falciparum* isolate with PfRh4 expression, as determined by immunofluorescence microscopy. Order of panels: phase, DAPI stain (labels nucleus), anti-Ron4-Alexa 488, anti-Rh4-Alexa-597, overlay. (b) Western blots showing expression of PfRh4 by *P. falciparum* isolates recently adapted to in vitro culture. Proteins were extracted from schizonts and the membrane was probed with anti-PfRh4 antibodies (upper panel) and anti-HSP70 antibodies (lower panel) as a loading control. The positions of molecular weight standards are indicated on the left. Isolates tested were 3D7, XIE (from PNG), Pf2004 (Ghana), Pf2006 (Ghana), HCS3 (Thailand) and a W2mef-PfRh4 knockout line (Rh4ko).

To determine the extent of PfRh4 expression and its potential as an immune target, we assessed PfRh4 protein expression among isolates infecting individuals in the cohort by immunofluorescence microscopy. To maintain the original phenotype, *P.falciparum* isolates were only cultured *in vitro* until schizont-stages were prevalent, but were not cultured through new cycles of invasion and replication. Laboratory adapted isolates 3D7 and HB3 were used as positive controls, with a PfRh4 knockout mutant as a negative control (the monoclonal antibody to PfRh4 did not label the PfRh4-knockout parasite line by IFA or western blot. We also stained for RON4, another rhoptry protein, to verify the location of PfRh4 staining. We found that 15 out of 22 isolates from infected children showed expression of PfRh4 proteins and were classified as positive (Table 4; Figure 3a). This suggests that PfRh4 is an important invasion ligand in this population. Previous reports of laboratory-adapted isolates show that 73% express PfRh4 (Supporting data, Table S2). Testing four recently adapted isolates [38] of different geographic origins by Western Blot, we found that isolates XIE010 (origin - PNG) and Pf2004 (Ghana) showed PfRh4 expression, whereas Pf2006 (Ghana) and HCS3 (Thailand) were PfRh4-negative (Figure 3b).

**Table 4:** Expression of PfRh4 in clinical isolates from PNG.

Isolate	Number of schizonts counted	Proportion PfRh4 positive (%)	PfRh4 expression
A	32	31.3	+
B	5	20	unclear
C	23	56.5	+
D	39	46.1	+
E	15	46.7	+
F	12	−	−
G	21	57	+
H	16	81.3	+
I	16	6.25	unclear
J	15	66.7	+
K	31	87.7	+
L	10	90	+
M	10	90	+
N	37	−	−
O	15	−	−
P	20	30	+
Q	24	41.7	+
R	52	34.6	+
S	7	−	−
T	28	−	−
U	54	77.8	+
V	61	50.8	+
3D7	117	40.1	+

Notes: Results from immunofluorescence assays using blood smears of *P. falciparum* isolates from infected children. Isolates A–V were stained with DAPI and anti-PfRh4 antibody in order to investigate the rate of PfRh4 expression in clinical isolates. % Rh4 positive: proportion of DAPI-positive schizonts that were also PfRh4-positive. An arbitrary cut-off for positivity was set at 3 DAPI-positive schizonts that were also Rh4-positive. As a reference, schizonts of isolate 3D7 were stained with anti-PfRh4 antibody in 4 independent experiments to determine the average percentage of positive PfRh4 staining (mean 40.1%, range of 20.7%).

### Limited Sequence Polymorphism in PfRh4

To further understand the importance of PfRh4 as an immune target and its potential as a vaccine candidate, we sequenced the regions coding for PfRh4.2 and PfRh4.9 in clinical isolates obtained in the population (Table 5). In the region of PfRh4.2, we found no SNPs among 13 isolates from the cohort and 16 isolates from diverse geographic origins. For PfRh4.9, we determined sequences from 12 isolates in the cohort; there were four SNPs resulting in one synonymous and 3 non-synonymous changes. In 11 sequences from public databases (plus isolate HCS3) there were 5 additional polymorphic sites, (1 synonymous, 4 non-synonymous). The 3 non-synonymous changes in the cohort sequences were also found in sequences from the databases. Furthermore, 11 of the sequences were identical to the reference strain 3D7, and 10 sequences differed at only 1 or 2 sites. The average nucleotide diversity (π) was 0.00095, and there was no departure from neutrality was detected by Tajima’s D value for population data (n = 12) or the Hudson-Kreitman-Aguade ratio for all data (n = 23) [44]. Thus, there is no evidence of significant selective pressure in the N-terminal region of PfRh4.

**Table 5:** Polymorphisms within regions of PfRh4.

		N	bp	SNPs	h	S	NS
I	PfRh4.2	13	525	0	1	0	0
	PfRh4.9	12	1817	4	4	1	3
II	PfRh4.2	16	525	0	1	0	0
	PfRh4.9	11	1648–1817*	8	6	1	7
III	PfRh4.2	29	525	0	1	0	0
	PfRh4.9	23	1648–1817*	9	7	2	7

Notes: Sequence analysis of PfRh4.2 and PfRh4.9 in clinical isolates derived from the Mugil population (I), global sequences of diverse geographic origins (II) and the combined datasets (III). For global sequences, we sequenced isolate HCS3, and obtained sequences for 3D7, Dd2, HB3, 7G8, FVO, CS2, D6, FCC2, IGH-CR14, K1, Pf_Senegal, SL and VS/1, IT line and Ghana from the Broad Institute and Sanger Institute public databases. N: number of isolates tested. bp: length of fragment sequenced in bp (PfRh4.2: bp 3829–4353; PfRh4.9: bp 170–1987). *: length of fragment varied between 1648bp and 1817 bp. SNPs: total number of single nucleotide polymorphisms observed in all sequences. h: total number of haplotypes observed. S: total number of synonymous mutations in all sequences. NS: total number of non-synonymous mutations in all sequences.

## Discussion

These findings represent a significant advance towards identifying targets of human immunity to malaria. Our broad evaluation of PfRh4 combining prospective studies of associations between antibodies and protective immunity, the functional activity of human antibodies, and PfRh4 expression by *P. falciparum* in the population provide important evidence, for the first time, suggesting that PfRh4 is a target of human immunity.

IgG1 and IgG3 were the predominant subclasses for antibodies to PfRh4, with very little IgG2 or IgG4, as observed for other merozoite antigens [25], [30], [31], [45]. High levels of IgG3 to PfRh4.9, containing the erythrocyte-binding region, were very strongly associated with reduced malaria risk; this is the strongest association with protection against malaria that we have observed in this cohort [25], [30], [31]. Importantly, this protective association remained strong and significant after adjusting for potential confounding factors of age, residential location, and parasitemia status at enrolment. High levels of IgG3 to PfRh4.2 were also strongly associated with protection against clinical malaria and high-density parasitemia, but this association was not as strong as that seen for IgG3 to PfRh4.9. Interestingly, IgG1 to PfRh4.9 was not significantly associated with protective immunity, suggesting that IgG3 might be functionally more important. Although a detailed knowledge of the functions of IgG subclasses in humans is lacking, prior studies report important differences in the function of IgG1 and IgG3 in other systems (eg. [46]), and known structural differences between IgG1 and IgG3 may influence specificity and function. Animal studies indicate that different IgG subclasses have different immunologic activities and roles, and lead to different outcomes [43]. Further studies are needed to understand the functional differences between IgG1 and IgG3 to PfRh4 and other merozoite antigens. The association between high levels of antibodies and protection from high-density parasitemia, but not re-infection per se, is consistent with PfRh4 antibodies acting by inhibition of erythrocyte invasion and parasite replication, which we observed *in vitro*. Human immunity to malaria is almost certainly mediated by responses to multiple target antigens, but few of these target antigens have been identified or studied in detail. Data presented here provide important evidence that antibodies to PfRh4 contribute to the protective immune response in humans. We have previously reported that antibodies to the EBAs and PfRh2 [25], [30], [31] are strongly associated with protection; collectively, these studies identify the EBA and PfRh invasion ligand families as important targets of immune responses in humans that appear to contribute to protection against malaria. Given that *P. falciparum* can vary the use and expression of EBA and PfRh4 invasion ligands as a possible means of immune evasion [7], it is likely that functional antibodies to multiple invasion ligands would be needed to provide effective immunity.

Demonstrating the functional significance of PfRh4 antibodies, affinity-purified human antibodies against the PfRh4.9 protein, which contains the erythrocyte-binding region, were potent inhibitors of SA-independent invasion, which requires the PfRh4 ligand. To our knowledge, this is the first report of human IgG to PfRh4 being invasion-inhibitory and one of the strongest lines of evidence that human antibodies specific to an invasion ligand may play an important role in immunity. Comparisons between antibody levels to PfRh4.9 in samples from children and the concentration of affinity-purified antibodies for invasion inhibition suggested that protected children have sufficiently high antibody levels to have inhibitory activity against *P. falciparum*. These data complement our findings of an association between antibodies to PfRh4 and protection from malaria, providing evidence that PfRh4 is a target of protective antibodies. In contrast, human PfRh4.2 antibodies did not inhibit invasion of erythrocytes. Hence, the functional relevance of PfRh4.2 antibodies is unclear, and they may have a different mode of action, such as interactions with Fc-receptors on immune cells or with complement. Alternatively, antibodies to PfRh4.2 may be a marker of immunity, or of responses to PfRh4 more broadly, rather than directly contributing to immunity. Presently, the acquisition of human growth-inhibitory antibodies to merozoite antigens has only been demonstrated for AMA1 and MSP1-19 [47], [48], and with some evidence supporting EBA175 as an additional target [7]. Studies have not previously related the antibody levels required for functional activity to levels present in protected individuals as we have done. Prior studies suggest that MP1-19 is not a major target of inhibitory antibodies in this population [49].

Most isolates infecting children demonstrated PfRh4 protein expression, consistent with our data suggesting PfRh4 may be an important target of acquired immunity. There are no prior reports of PfRh4 protein expression in clinical isolates, but two studies of African children found that *pfrh4* gene expression was detected in a minority of isolates [19], [20]. Differences between these studies and ours may represent population-specific differences, or differences in the sensitivity of the methods used. Consistent with our studies on clinical isolates, most culture adapted isolates (from diverse geographic origins) show expression of PfRh4 protein. Further studies are needed to assess a larger number of isolates in different populations and relationship between PfRh4 expression and acquired antibodies. Sequence analysis identified little polymorphism in the PfRh4 erythrocyte-binding region and no polymorphisms in the PfRh4.2 region. In contrast, PfRh2 has a highly polymorphic stretch in the N-terminal region [30] that encodes the erythrocyte-binding domain [50], [51]. It is possible the variable expression of PfRh4 is used a means of immune evasion [7], rather than immune evasion via the evolution of polymorphisms. The limited polymorphism in PfRh4 may make it attractive as a vaccine candidate, especially when partnered with other antigens, such as EBA175, to block the use of alternate invasion pathways [7], [52]. PfRh5 also has limited polymorphisms [53], and a recent report suggested that there was minimal acquisition of IgG to recombinant PfRh5 in a population of malaria-exposed Kenyans [54]. One study reported that expression of the *pfrh5* gene was variable among *P. falciparum* isolates in The Gambia [20], as seen for other *pfrh* genes, whereas other data suggests PfRh5 protein is expressed by all laboratory-adapted isolates [53], [54]. Our preliminary studies suggest that the prevalence of antibodies to recombinant PfRh5 is high in our PNG study population (J. Richards, J. Beeson, unpublished observations). Further studies to understand the expression of PfRh ligands and the acquisition of antibodies to them will be valuable for understanding the importance of PfRh proteins as targets of immunity and their potential for vaccine development.

Understanding the targets of acquired human immunity is valuable for several reasons. One of the important criteria for objectively prioritizing antigens for vaccine development against malaria, as with other infectious pathogens, is the demonstration that immune responses to that antigen are associated with protection from malaria [22], [23]. As noted earlier, there is presently a major deficiency in our knowledge of human immune responses to the many potential blood-stage antigens of human malaria. Furthermore, our level of understanding human immune responses to complex pathogens is very limited. Knowledge of immunity to infectious diseases is largely based on studies of much simpler organisms (viruses and some bacteria) that have much smaller genomes than complex organisms such as *P. falciparum*, and have few target antigens. Often this knowledge also relies heavily on principles established in small animal models that may not be wholly representative of human disease and immune responses. There is increasing interest in using serological assays as a low cost tool for surveillance of malaria exposure in populations to guide control efforts by identifying populations at risk, and evaluating the impact of malaria control interventions on malarial immunity, and this is arising as a major potential application of a detailed knowledge of human immunity to malaria. Identifying key antibody targets would advance these strategies and facilitate the development of biomarkers to monitor immunity in populations.

In conclusion, our findings suggest PfRh4 is a target of human antibodies that contribute to protective immunity, and PfRh4 may warrant further consideration in malaria vaccine development. The PfRh ligands are not present in rodent or simian malarias and, therefore, responses to PfRh ligands cannot be easily studied in animal models. This emphasizes the importance of human studies to evaluate these antigens as targets of protective immunity and aid the identification and prioritization of candidates for vaccine development. Furthermore, the broad approach used here to evaluate PfRh4 as an immune target could serve as a valuable framework to assess and prioritize numerous other merozoite antigens being identified in the post-genomic era as immune targets and potential vaccine candidates.

## Supporting Information

Table S1 **Association between antibodies and risk of high-density parasitemia.** Study participants were stratified into 3 equal groups according to low, medium or high levels of antigen-specific antibodies. Hazard ratios were calculated comparing those with high versus low levels of antibodies (HvL) and medium versus low levels (MvL) of antibodies for the risk of high-density parasitemia (>5000 parasites/µl blood) over 6 months of follow-up; analysis was based on first episode only. Unadjusted hazard ratios (uHR), and adjusted (age-adjusted and location-adjusted) hazard ratios hazard ratios (aHR) were calculated.
(DOCX)

Table S2 **Expression of PfRh4 by laboratory-adapted isolates.** Note on isolates: *Isolates W2mef and Dd2 are thought to be genetically identical; ^#^ isolates FCR3 and E8B (a clone of the IT line) are thought to be genetically identical. References: Tham WH, Schmidt CQ, Hauhart RE, Guariento M, Tetteh-Quarcoo PB, et al. (2011). Plasmodium falciparum uses a key functional site in complement receptor type-1 for invasion of human erythrocytes. Blood 118∶1923–1933; Stubbs J, Simpson K, Triglia T, Plouffe D, Tonkin C, et al. (2005), Molecular mechanism for switching of P. falciparum invasion pathways into human erythrocytes. Science 309∶1384–1387; Gaur D, Furuya T, Mu J, Jiang LB, SuXZ, et al. (2006) Upregulation of expression of the reticulocyte homology gene 4 in the Plasmodium falciparum clone Dd2 is associated wit a switch in the erythrocyte invasion pathway. Molec Biochem Parasitol 145∶205–215.
(DOCX)
