# Supplementary material for: The Plasmodium falciparum Erythrocyte Invasion Ligand Pfrh4 as a Target of Functional and Protective Human Antibodies against Malaria
Source: PLoS One. 2012 Sep 20;7(9):e45253. doi: 10.1371/journal.pone.0045253 (PMC3447948; doi:10.1371/journal.pone.0045253)
Supplement: Table S1 — Association between antibodies and risk of high-density parasitemia. Study participants were stratified into 3 equal groups according to low, medium or high levels of antigen-specific antibodies. Hazard ratios were calculated comparing those with high versus low levels of antibodies (HvL) and medium versus low levels (MvL) of antibodies for the risk of high-density parasitemia (>5000 parasites/µl blood) over 6 months of follow-up; analysis was based on first episode only. Unadjusted hazard ratios (uHR), and adjusted (age-adjusted and location-adjusted) hazard ratios hazard ratios (aHR) were calculated. (DOCX) [file pone.0045253.s001.docx]

**Table S1: Association between antibodies and risk of high-density parasitemia**

| Antigen |  | uHR | p | uHR | p | aHR | p | aHR | p |
| --- | --- | --- | --- | --- | --- | --- | --- | --- | --- |
|  |  | MvL |  | HvL |  | MvL |  | HvL |  |
|  |  | [95% CI] |  | [95% CI] |  | [95% CI] |  | [95% CI] |  |
| Rh4.2 | IgG | 0.69 | 0.14 | 0.4 | 0.002 | 0.84 | 0.5 | 0.51 | 0.03 |
|  |  | [0.42-1.13] |  | [0.23-0.71] |  | [0.5-1.4] |  | [0.28-0.93] |  |
|  | IgG1 | 0.61 | 0.1 | 0.64 | 0.1 | 0.66 | 0.13 | 0.71 | 0.22 |
|  |  | [0.36-1.04] |  | [0.38-1.09] |  | [0.38-1.13] |  | [0.41-1.23] |  |
|  | IgG3 | 0.57 | 0.03 | 0.39 | 0.001 | 0.69 | 0.16 | 0.52 | 0.03 |
|  |  | [0.34-0.95] |  | [0.22-0.68] |  | [0.41-1.16] |  | [0.29-0.93] |  |
| Rh4.9 | IgG | 0.45 | 0.02 | 0.77 | 0.39 | 0.49 | 0.04 | 0.78 | 0.42 |
|  |  | [0.23-0.9] |  | [0.43-1.4] |  | [0.25-0.98] |  | [0.43-1.44] |  |
|  | IgG1 | 0.61 | 0.12 | 0.67 | 0.21 | 0.72 | 0.31 | 0.72 | 0.32 |
|  |  | [0.33-1.15] |  | [0.36-1.25] |  | [0.38-1.36] |  | [0.38-1.36] |  |
|  | IgG3 | 0.42 | 0.01 | 0.34 | 0.001 | 0.53 | 0.05 | 0.53 | 0.08 |
|  |  | [0.23-0.78] |  | [0.18-0.66] |  | [0.28-0.99] |  | [0.26-1.07] |  |

**Notes:**

Study participants were stratified into three equal groups according to low, medium or high levels of antigen-specific antibodies. Hazard ratios were calculated comparing those with high versus low levels of antibodies (HvL) and medium versus low levels (MvL) of antibodies for the risk of high-density parasitemia (>5000parasites/µl blood) over 6 months of follow-up; analysis was based on first episode only. Unadjusted hazard ratios (uHR), and adjusted (age-adjusted and location-adjusted) hazard ratios hazard ratios (aHR) were calculated.

**Table S2: Expression of PfRh4 by laboratory-adapted isolates**

| **Isolate** | **Rh4 expression (method)** | **reference** |
| --- | --- | --- |
| 3D7 | yes (Western blot) | Tham et al., 2011 |
| D10 | yes (Western blot) | Tham et al., 2011 |
| W2mef* | no (Western blot) | Stubbs et al., 2005 |
| E8B^#^ | yes (Western blot) | Tham et al., 2011 |
| CSL2 | no (Western blot) | Tham et al., 2011 |
| T994 | yes (Western blot) | Tham et al., 2011 |
| FCR3^#^ | yes (Western blot) | Tham et al., 2011 |
| 7G8 | yes (Western blot) | Tham et al., 2011 |
| HB3 | yes (Western blot) | Tham et al., 2011 |
| MCamp | yes (Western blot) | Tham et al., 2011 |
| Dd2* | no (Western blot) | Gaur et al., 2006 |

**Notes:**

*Isolates W2mef and Dd2 are thought to be genetically identical; ^#^ Isolates FCR3 and E8B (a clone of the IT line) are thought to be genetically identical.

References:

Tham WH, Schmidt CQ, Hauhart RE, Guariento M, Tetteh-Quarcoo PB, et al. (2011) Plasmodium falciparum uses a key functional site in complement receptor type-1 for invasion of human erythrocytes. Blood 118: 1923-1933.

Stubbs J, Simpson K, Triglia T, Plouffe D, Tonkin C, et al. (2005) Molecular mechanism for switching of P. falciparum invasion pathways into human erythrocytes. Science 309: 1384-1387.

Gaur D, Furuya T, Mu J, Jiang LB, Su XZ, et al. (2006) Upregulation of expression of the reticulocyte homology gene 4 in the Plasmodium falciparum clone Dd2 is associated with a switch in the erythrocyte invasion pathway. Molecular & Biochemical Parasitology 145: 205-215.
