# Supplementary material for: The Plasmodium falciparum Erythrocyte Invasion Ligand Pfrh4 as a Target of Functional and Protective Human Antibodies against Malaria
Source: PLoS One. 2012 Sep 20;7(9):e45253. doi: 10.1371/journal.pone.0045253 (PMC3447948; doi:10.1371/journal.pone.0045253)
Supplement: Table S2 — Expression of PfRh4 by laboratory-adapted isolates. Note on isolates: *Isolates W2mef and Dd2 are thought to be genetically identical; # isolates FCR3 and E8B (a clone of the IT line) are thought to be genetically identical. References: Tham WH, Schmidt CQ, Hauhart RE, Guariento M, Tetteh-Quarcoo PB, et al. (2011). Plasmodium falciparum uses a key functional site in complement receptor type-1 for invasion of human erythrocytes. Blood 118∶1923–1933; Stubbs J, Simpson K, Triglia T, Plouffe D, Tonkin C, et al. (2005), Molecular mechanism for switching of P. falciparum invasion pathways into human erythrocytes. Science 309∶1384–1387; Gaur D, Furuya T, Mu J, Jiang LB, SuXZ, et al. (2006) Upregulation of expression of the reticulocyte homology gene 4 in the Plasmodium falciparum clone Dd2 is associated wit a switch in the erythrocyte invasion pathway. Molec Biochem Parasitol 145∶205–215. (DOCX) [file pone.0045253.s002.docx]

**Table S2: Expression of PfRh4 by laboratory-adapted isolates**

| **Isolate** | **Rh4 expression (method)** | **reference** |
| --- | --- | --- |
| 3D7 | yes (Western blot) | Tham et al., 2011 |
| D10 | yes (Western blot) | Tham et al., 2011 |
| W2mef* | no (Western blot) | Stubbs et al., 2005 |
| E8B^#^ | yes (Western blot) | Tham et al., 2011 |
| CSL2 | no (Western blot) | Tham et al., 2011 |
| T994 | yes (Western blot) | Tham et al., 2011 |
| FCR3^#^ | yes (Western blot) | Tham et al., 2011 |
| 7G8 | yes (Western blot) | Tham et al., 2011 |
| HB3 | yes (Western blot) | Tham et al., 2011 |
| MCamp | yes (Western blot) | Tham et al., 2011 |
| Dd2* | no (Western blot) | Gaur et al., 2006 |

Note on isolates:

*Isolates W2mef and Dd2 are thought to be genetically identical; ^#^ Isolates FCR3 and E8B (a clone of the IT line) are thought to be genetically identical.

References:

Tham WH, Schmidt CQ, Hauhart RE, Guariento M, Tetteh-Quarcoo PB, et al. (2011) Plasmodium falciparum uses a key functional site in complement receptor type-1 for invasion of human erythrocytes. Blood 118: 1923-1933.

Stubbs J, Simpson K, Triglia T, Plouffe D, Tonkin C, et al. (2005) Molecular mechanism for switching of P. falciparum invasion pathways into human erythrocytes. Science 309: 1384-1387.

Gaur D, Furuya T, Mu J, Jiang LB, Su XZ, et al. (2006) Upregulation of expression of the reticulocyte homology gene 4 in the Plasmodium falciparum clone Dd2 is associated with a switch in the erythrocyte invasion pathway. Molecular & Biochemical Parasitology 145: 205-215.
